# Mechanisms Linking Obesity, Insulin Resistance, and Alzheimer’s Disease: Effects of Polyphenols and Omega-3 Polyunsaturated Fatty Acids

**DOI:** 10.3390/nu17071203

**Published:** 2025-03-29

**Authors:** Mahsa Yavari, Nishan Sudheera Kalupahana, Breanna N. Harris, Latha Ramalingam, Yujiao Zu, Chanaka Nadeeshan Kahathuduwa, Naima Moustaid-Moussa

**Affiliations:** 1 Department of Nutritional Sciences, Texas Tech University, Lubbock, TX 79409, USA; mahsa_yavari@hsph.harvard.edu (M.Y.); lramalin@syr.edu (L.R.); yujiao.zu@ttu.edu (Y.Z.); 2 Obesity Research Institute, Office of Research & Innovation, Texas Tech University, Lubbock, TX 79409, USA; breanna.n.harris@ttu.edu (B.N.H.); chanaka.kahathuduwa@ttuhsc.edu (C.N.K.); 3 Department of Nutrition and Health, College of Medicine and Health Sciences, United Arab Emirates University, Al Ain P.O. Box 15551, United Arab Emirates; nkalupahana@uaeu.ac.ae; 4 Department of Biological Sciences, Texas Tech University, Lubbock, TX 79409, USA; 5 Institute for One Health Innovation, Offices of Research & Innovation, Texas Tech University, Texas Tech Health Sciences Center, Lubbock, TX 79409, USA; 6 Department of Neurology, Texas Tech University Health Sciences Center, El Paso, TX 79409, USA

**Keywords:** Alzheimer’s disease, obesity, diabetes, insulin resistance, polyphenols, omega-3 polyunsaturated fatty acids

## Abstract

Alzheimer’s disease (AD) is a complex neurodegenerative disorder characterized by progressive cognitive decline, memory loss, and behavioral changes. It poses a significant global health challenge. AD is associated with the accumulation of amyloid-β (Aβ) plaques and neurofibrillary tangles (NFTs) in the brain, along with chronic inflammation, dysfunctional neurons, and synapse loss. While the prevalence of AD continues to rise, the current FDA-approved drugs offer only limited effectiveness. Emerging evidence suggests that obesity, insulin resistance (IR), and type 2 diabetes mellitus (T2DM) are also implicated in AD pathogenesis, with epidemiological studies and animal models confirming the impact of IR on Aβ accumulation, and high-fat diets also exacerbating Aβ accumulation. Since neuroinflammation activated by Aβ involves the nuclear factor kappa-light-chain-enhancer of the activated B cell (NF-κB) pathway, the inhibition of NF-κB and NLRP3 inflammasome activation are potential therapeutic strategies in AD. Bioactive compounds, including polyphenols (resveratrol, epigallocatechin-3-gallate, curcumin, and quercetin), and omega-3 polyunsaturated fatty acids, show promising results in animal studies and clinical trials for reducing Aβ levels, improving cognition and modulating the signaling pathways implicated in AD. This review explores the interplay between obesity, IR, inflammation, and AD pathology, emphasizing the potential of dietary compounds and their role in reducing inflammation, oxidative stress, and cognitive decline, as viable strategies for AD prevention and treatment. By integrating epidemiological findings, observational studies, and clinical trials, this review aims to provide a comprehensive understating of how metabolic dysfunctions and bioactive compounds influence AD progression.

## 1. Introduction

Alzheimer’s disease (AD) is a chronic and progressive neurodegenerative disorder characterized by a predominant impairment of anterograde episodic memory [1]. Currently, more than 55 million people are living with AD worldwide, which includes 6 million Americans, and in the US, by 2050, this number is predicted to reach around 13 million [2]. Due to its high prevalence, it accounts for two-thirds of all dementia diagnoses [2,3]. AD primarily manifests as dementia that often affects memory, language, and other cognitive functions and results in the loss of functional abilities [4]. It is also associated with depression, insomnia [5,6], olfactory impairment [7], and macro- or micro-nutrient deficiencies [8]. The standard clinical diagnosis of AD includes the presence of (1) large extracellular deposits or plaques of amyloid-β (Aβ) peptides outside the neuronal cells in different regions of the brain (especially in the cortex and hippocampus) and (2) neurofibrillary tangles (NFTs) inside the neuronal cells that are composed primarily of hyperphosphorylated tau protein [9,10].

**Figure 1:**
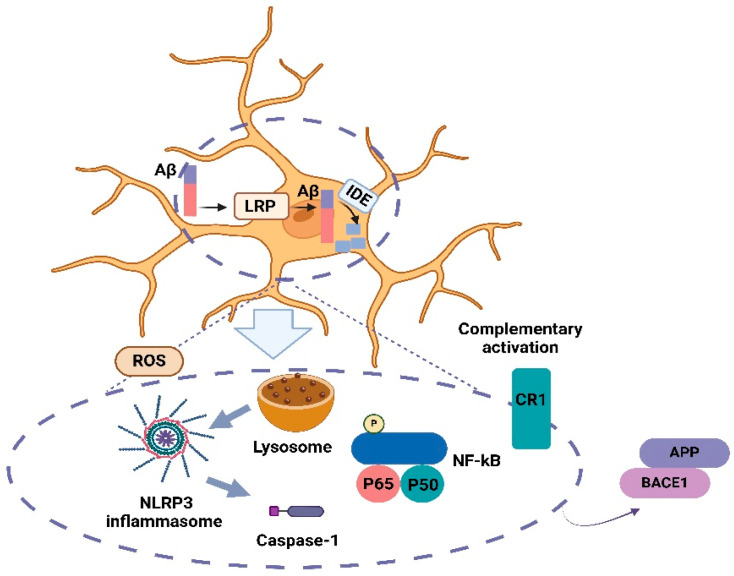
Brain microglia activation in AD. Microglia are effective in Aβ clearance, neuroinflammation, and the production and aggregation of Aβ. Also, an Aβ pathologic increase in the brain and the formation of plaques can increase the recruitment of microglia around the plaques and trigger inflammatory signaling in the positive feedback cycle. APP, amyloid precursor protein; Aβ, amyloid-beta; BACE1, beta-secretase 1; CR1, complement receptor 1; IDE, insulin-degrading enzyme; LRP, low-density lipoprotein receptor-related protein 1; NF-κB, nuclear factor kappa; P, phosphorylated; ROS, reactive oxygen species.

Aβ is produced by the sequential cleavage of the amyloid precursor protein (APP) by β- and γ-secretase in the amyloidogenic pathway [11]. β-secretase (BACE1) cleavage in the extracellular domain and γ-secretase cleavage in the transmembrane region are necessary to release Aβ from the precursor molecule [12]. While the majority of Aβ peptides generated by γ-secretase activity end at amino acid 40 (Aβ40), a small proportion ends at amino acid 42 (Aβ42) [13]. The accumulation of Aβ in the brain, a hallmark of AD, is influenced by the balance between its production and clearance [6]. Aβ clearance mechanisms include transport via lipoprotein receptor-related protein 1 (LRP1), degradation by enzymes like insulin-degrading enzyme (IDE), and phagocytosis by microglia [14]. LRP1 facilitates Aβ transport across the blood–brain barrier and reduces Aβ production by downregulating BACE1 [15,16,17,18]. IDE degrades both Aβ and insulin, while microglia, the brain’s primary immune cells, clear Aβ through phagocytosis [15,18,19]. Microglia, the primary immune cells in the central nervous system, play a crucial role in AD by Aβ deposits and soluble Aβ through phagocytic activity [20]. NF-κB signaling and NOD-, LRR-, and pyrin domain-containing protein 3 (NLRP3) inflammasome activation are key inflammatory pathways involved in AD, with studies showing that inhibiting these pathways can reduce inflammation and oxidative stress, potentially improving therapeutic outcomes [21] (Figure 1).

AD shares key pathological mechanisms with obesity and insulin resistance (IR), including chronic inflammation, oxidative stress, and impaired metabolic signaling [22]. Obesity and IR contribute to amyloidogenic pathways by promoting beta-amyloid aggregation and tau hyperphosphorylation, exacerbating neuroinflammation and neuronal dysfunction [23]. Given these shared mechanisms, targeting metabolic dysregulation through nutritional interventions presents a promising strategy for AD prevention. On the other hand, when investigating the pathological mechanisms of AD, selecting an AD mouse model is a critical factor that can significantly influence the outcomes. Given the heterogeneity of AD pathology and its complex relationship with metabolic dysfunction, different transgenic mouse models [24,25,26] present unique advantages and limitations. Each model exhibits distinct pathological mechanisms, including variations in Aβ deposition, tau pathology, neuroinflammation, and metabolic dysfunction, which can lead to inconsistencies in study outcomes when assessing the impact of obesity and systemic inflammation on AD progression.

In this review, we summarize findings from both clinical and preclinical studies, highlighting key considerations for designing mouse model experiments that accurately reflect the metabolic–inflammatory interactions driving AD progression. Additionally, we explore the role of dietary components in modulating amyloid pathology and inflammation, highlighting their potential to mitigate AD risk by addressing obesity- and IR-related pathways. Ultimately, the objective of this review is to integrate epidemiological, observational, and published and ongoing clinical and preclinical studies to explore the pathophysiological mechanisms of AD, focusing on neuroinflammation, Aβ accumulation, and metabolic dysfunction, particularly the roles of obesity, IR, and T2DM in AD progression. We further aim to explore the therapeutic potential of bioactive compounds (polyphenols and omega-3 fatty acids) in modulating neuroinflammation, oxidative stress, and cognition to identify dietary and anti-inflammatory interventions for AD prevention and treatment.

### 1.1. Obesity, Chronic Inflammation, and AD

Obesity is a complex disease characterized by excess accumulation of body fat that presents a risk to health. [27]. Many studies have shown a chronic low-grade inflammation in adipose tissue (AT) in people with obesity [28], which is associated with metabolic derangements of tissues [29]. The inflammation of adipocytes is accompanied by the release of pro-inflammatory cytokines, which interfere with the normal functioning of the AT and other tissues [30,31]. The initial report for obesity-related inflammation was from a study of elevated tumor necrosis factor (TNFα) in AT in an obese animal model [32].

Cytokines facilitate the passage of leukocytes through the blood–brain barrier (BBB) into the central nervous system (CNS), leading to synaptic loss, hypothalamic dysfunction, cognitive impairment, and neurodegeneration [33]. The passage of cytokines through the BBB from peripheral tissues to the brain has been demonstrated by various research groups [34,35]. Meta-analyses have shown that individuals with obesity have twice the risk of developing AD compared to those with a normal body mass index (BMI) [36,37]. Additionally, genetic studies have established a link between midlife obesity [38,39] supported by multiple clinical (Table 1) and preclinical studies (Table 2). A high-fat diet (HFD) consumption contributes to obesity, inflammation, and oxidative stress, resulting in memory deficits in both humans and transgenic mouse models of AD [40,41]. Adipose tissue in individuals with obesity secretes pro-inflammatory adipokines and cytokines, such as leptin, IL-1β, and IL-6 [32]. This peripheral inflammation then activates endothelial and glial cells, particularly microglia, promoting the production of brain cytokines [42]. Evidence has shown that amyloid-driven models such as APP/PS1, Tg2576, and 5xFAD exhibit increased Aβ deposition, oxidative stress, and neuroinflammation in response to DIO, with Tg2576 mice particularly prone to IR [43]. By contrast, tau-inclusive models like 3xTg-AD show accelerated tau hyperphosphorylation and synaptic dysfunction under DIO, highlighting obesity’s role in tau-driven neurodegeneration [25]. Additionally, the genetic background also modulates metabolic responses, with mouse models with a B6 background exhibiting stronger microglial activation, while 129S1/SvImJ-based models like 3xTg-AD demonstrate more severe tau-related neurodegeneration under inflammatory conditions [44]. These findings highlight the complex relationship between obesity, inflammation, and AD risk. While obesity-driven inflammation exacerbates neurodegeneration, the metabolic profile of an individual may influence disease progression. Understanding these mechanisms is crucial for developing targeted dietary interventions that mitigate the detrimental effects of obesity while preserving any potential protective factors against AD.

On the other hand, a recent study using data from the Chinese Alzheimer’s Biomarker and Lifestyle database found that metabolically healthy obesity and lipids may provide a protective effect against neurodegenerative changes and AD in late life. Among 604 cognitively normal participants, obese individuals had lower cerebrospinal fluid (CSF) total tau and phosphorylated tau levels compared to non-obese individuals [45]. Similar findings were reported by a large cohort study of 12.3 million people and a study using the AD Neuroimaging Initiative database, suggesting that metabolically healthy obesity in late life was associated with a lower risk of dementia [46]. The above observations can be explained by several mechanisms. These include increased levels of adipose-related hormones, such as insulin-like growth factor I [47] and leptin [48], which may exert neurotrophic and synaptic plasticity effects in the hippocampus, supporting better cognitive performance. Additionally, metabolically healthy obese individuals tend to have more lean body mass and less fat mass compared to metabolically unhealthy obese individuals [49], who exhibit greater central adiposity and inflammation. Given these complexities and the key distinction of chronic inflammation between metabolically unhealthy and healthy obesity, further research is needed to explore the role of lipids, metabolic states, and genetic factors in AD progression.

**Table 1:** Summary of clinical studies linking AD and obesity.

Study	Subjects	Findings	Ref
Launceston study Case–control study	50 elderly patients with AD, male and female	Obesity (BMI > 30 kg/m^2^), abdominal obesity (waist-to-hip ratio (WHR): >0.8 (women), >0.9 (men)) were associated with AD	[50]
New York longitudinal study	Male and female, without dementia at baseline, 145 AD patients at the end	WHR was correlated with higher risk of AD	[51]
The HUNT Study Longitudinal study	654 male and female patients with AD	Significant associations between AD and obesity	[52]
AD Neuroimaging Initiative and the Cardiovascular Health Study-Cognition Study. Observational study	700 male patients with MCI or AD	A higher BMI was associated with brain volume deficits in frontal, temporal, parietal, and occipital lobes measured by MRI	[53]

**Table 2:** Animal studies investigating the association of AD and obesity.

AD Model	Intervention	Findings	Ref
APP/PS1 male and female	45% HFD 8 and 12 months	HFD resulted in increased food intake, body weight and glucose intolerance, inflammation, and tau and Aβ in the cortex	[54]
Tg2576 AD male	60% HFD 7 months	HFD resulted in IR, higher BW, higher Aβ plaques, and GSK	[55]
Tg2576 AD female	42% HFD 4–16 weeks	Increased food intake, body weight, and Aβ content in the brain	[56]
APP/PS1 TG male	High-sucrose diet 10 to 20 or 28 weeks	Higher BW and fat mass, leptin resistance, hyperinsulinemia	[57]
3xTg-CD AD male and female	60% HFD 2 months	HFD aggravated brain atrophy and memory impairments, impaired brain structural integrity	[58]
AppNL/NL knock-in male	60% HFD 10 months	HFD impaired hippocampal potentiation	[59]
APP/PS1 male and female	42% HFD 17 months	Poorer memory performance, impaired social interactions, and increases in Aβ monomers and plaques with HFD	[60]
AppNL−F/NL−F male mice	40% HFD 14 months	Decreased the expression of the Aβ-binding protein transthyretin and Aβ deposition in hippocampus	[61]

APP/PS1: amyloid precursor protein/presenilin 1; Tg2576: APP Swedish mutation (KM670/671NL); 3xTg: triple-transgenic (APP: KM670/671NL, PSEN1: M146V, and MAPT: P301L); AppNL: APP knock-in; HFD: high-fat diet.

### 1.2. Diabetes and AD

Epidemiological studies confirmed that obesity-related type two diabetes mellitus (T2DM) increased the risk of developing AD compared to non-obesity-related T2DM [62,63,64]. Transgenic AD mouse models have further validated this connection, demonstrating how IR and hyperinsulinemia contribute to AD pathogenesis [55,65]. Table 3 summarizes the human studies linking IR and AD. One key mechanism involves IDE, which breaks down both insulin and Aβ in the brain [66,67]. In a neuroblastoma cell model, IR caused inactivation of IDE, which can cause the accumulation of Aβ due to the failure of Aβ clearance [68]. Additionally, IR disrupts the non-amyloidogenic pathway by reducing α-secretase expression, leading to increased Aβ peptide production [69].

Beyond Aβ accumulation, IR and hyperinsulinemia also contribute to tau pathology. They activate the mitogen-activated protein kinase (MAPK) signaling pathway, upregulating BACE1 expression and increasing phosphatidylinositol 3-kinase (PI3K)/protein kinase B (AKT) and glycogen synthase kinase 3 (GSK3) activity, ultimately driving tau hyperphosphorylation and neurodegeneration [70,71].

Animal studies have provided further evidence of the link between IR and AD (Table 4). Wakabayashi et al. [72] found that after six months on an HFD, 9-month-old male A7-Tg mice exhibited increased body weight and reduced insulin sensitivity compared to those on a calorie-restricted diet. HFD-fed mice also had elevated Aβ levels and decreased clearance in CSF and plasma. Furthermore, peripheral insulin injections at 5 months of age led to lower insulin receptor expression compared to non-Tg controls [72]. Similarly, Velazquez et al. [73] assessed glucose tolerance in Tg2576 and 3xTg-AD mouse models at 10 and 16 months of age, revealing that older Tg mice exhibited peripheral IR and reduced IRS-1 activity compared to non-Tg mice [73]. Moreover, strain differences in peripheral metabolism and pancreatic function influence diabetes and insulin-resistant progression, with B6 background AD models, like 5xFAD and APP/PS1, showing a greater susceptibility to DIO and IR compared to 129S1 background models, like 3xTg-AD [74]. These findings highlight the crucial role of IR in AD pathogenesis, linking metabolic dysfunction to amyloid accumulation, tau hyperphosphorylation, and cognitive decline. Given the strong evidence from both human and animal studies, targeting IR through dietary and pharmacological interventions may offer a promising strategy to mitigate AD risk and progression.

**Table 3:** Human studies linking IR, type 2 diabetes mellitus, and AD.

Study	Subjects	Findings	Reference
The Rotterdam Study Prospective population-based cohort study	n = 6370 male and female 2.1 years follow-up	T2DM increased the risk of dementia and AD in elderly individuals; insulin-treated patients had the highest risk and T2DM-attributable risk of dementia by 8.8%	[75]
Observational study	n = 1262 male and female 4.3 years follow-up	Diabetes was associated with AD and cognitive impairment without dementia	[76]
Longitudinal cohort study	n = 824 male and female 5.5 years follow-up	T2DM was associated with developing AD, and the risk of incidence of AD was 65% higher in those with diabetes	[77]
Honolulu–Asia Aging Study Prospective cohort study	n = 2574 male	T2DM is a risk factor for AD, with the association being stronger among APOEε4 carriers	[78]
Israeli Ischemic Heart Disease Study Observational, longitudinal study	n = 1892 male	Confirmed diabetes as a risk factor for dementia	[79]

**Table 4:** In vivo studies linking IR, type 2 diabetes mellitus, and AD.

AD Model/Sex/Age	Intervention	Findings	Ref
B6-STZ/male/8–12 weeks	STZ-induced diabetic	Downregulation of LRP1 expression and its function of transporting Aβ across the BBB in diabetic mice	[80]
APP-STZ/male/8 months	STZ-induced diabetic	Mice with both APP overexpression and diabetes showed insulin receptor reduction and more phosphorylated tau and Aβ plaques	[81]
5xFAD-STZ/-/5 months	STZ-induced diabetic	STZ-induced insulin-deficient diabetes exacerbated Aβ accumulation by elevating expression levels of the β-secretase enzyme BACE1 and its substrate APP in the 5xFAD mouse model of AD	[82]
SAMP8/-/11 months	Metformin 20 mg/kgor 200 mg/kg subcutaneously (8 weeks)	Metformin improved learning and memory, decreased Aβ and tau proteins	[83]
3xTg Psen1tm1Mpm/male/12 months	-	Proteomics data showed altered O-GlcNAcylation levels in TG mice	[84]

STZ: streptozotocin; 5xFAD: five-familial (Swedish KM670/671NL, Florida I716V, London V717I, PSEN1: M146L and L286V).

### 1.3. Insulin Signaling in the Brain in AD

Building on the connection between obesity, IR, and AD, insulin dysfunction in the brain further exacerbates neurodegenerative processes. As discussed earlier, obesity-related metabolic disturbances, including chronic inflammation, increase AD risk by promoting Aβ accumulation and tau pathology. IR serves as a key mechanistic link, disrupting neuronal survival and synaptic plasticity.

After crossing the blood–brain barrier, insulin regulates cognition and metabolism through pathways such as PI3K/Akt and MAPK/ERK [85,86].

However, in AD, Aβ accumulation and neuroinflammation lead to elevated TNFα levels, activating stress kinases like c-Jun N-terminal kinase (JNK). This results in the inhibitory serine phosphorylation of IRS-1, impairing insulin signaling and reducing IDE activity, which further accelerates Aβ accumulation [87,88]. Additionally, increased GSK-3β activity due to insulin dysfunction contributes to tau hyperphosphorylation and neurodegeneration [88].

Animal studies provide strong evidence for the role of metabolic dysregulation in AD progression. A high-fat/high-sugar diet induced weight gain, peripheral IR, and impaired insulin receptor signaling in mice, accompanied by increased brain inflammation and the activation of JNK, MAPK, and NF-κB [87]. Notably, antidiabetic drugs such as metformin have been shown to counteract these effects by increasing p-AMPK and IDE levels in AD mouse models [89,90]. Further reinforcing this link, studies on transgenic AD mouse models indicate that an HFD reduces insulin receptor expression and an increase in Aβ accumulation. Female Tg2576 mice on an HFD exhibited greater hippocampal Aβ accumulation than chow-fed controls [91], while another study found elevated insulinemia, leptin, and Aβ40/Aβ42 expression in Tg2576 mice after prolonged HFD exposure [55]. Similarly, APPswe/PS1dE9 mice on an HFD showed significantly higher glucose, inflammatory cytokine, and Aβ levels compared to age-matched non-Tg controls [56]. These findings underscore the interplay between obesity, IR, and AD pathology, reinforcing the need for early metabolic intervention. Given the impact of nutrition on both insulin signaling and neuroinflammation, the following sections will explore dietary strategies that may mitigate amyloidogenic pathways and neuroinflammatory processes, offering potential preventive measures against AD.

### 1.4. Adipokines and AD

Expanding on the link between metabolic dysfunction and AD, adipokines, hormones secreted by adipose tissue, further modulate neurodegenerative pathways. Given that obesity promotes IR and inflammation, it is not surprising that altered adipokine signaling contributes to AD pathology.

Leptin, primarily secreted by white adipose tissue, plays a crucial role in energy homeostasis and brain function. At normal levels, it enhances synaptic function, learning, and memory [92]. However, obesity-induced leptin resistance disrupts these effects and is often accompanied by brain IR [93]. In AD, patients exhibit higher leptin and lower adiponectin levels compared to healthy individuals, suggesting metabolic dysregulation contributes to disease progression [94].

Leptin interacts with insulin signaling in the brain, particularly through pro-opiomelanocortin-expressing neurons, which regulate energy balance and glucose metabolism [95]. Experimental models demonstrate that leptin reduces beta-secretase activity and enhances APOE-dependent Aβ clearance, underscoring its neuroprotective role [96]. However, high-fat or high-sugar diets impair leptin signaling, exacerbating Aβ accumulation and inflammation in the brain. In APP/PS1 mice, diet-induced leptin resistance occurred even before plaque formation, correlating with early Aβ deposition [57,97]. Additionally, long-term Aβ exposure promotes hypothalamic leptin resistance, further contributing to metabolic dysfunction and weight gain [97]. Adiponectin, another key adipokine, exhibits anti-inflammatory and neuroprotective properties. In aged adiponectin-knockout mice, increased Aβ accumulation, tau phosphorylation, and impaired insulin signaling were observed, along with spatial memory deficits [98]. The interplay between obesity, IR, and AD is further complicated by adipokine dysregulation. Leptin and adiponectin influence Aβ metabolism, tau phosphorylation, and neuroinflammation, yet their protective functions are diminished in obesity and metabolic disorders. Given their overlapping pathways with insulin signaling, interventions targeting adipokine balance through dietary, pharmacological, or lifestyle strategies may offer new avenues for AD prevention and treatment.

### 1.5. Role of Bioactive Food Compounds in AD

**Figure 2:**
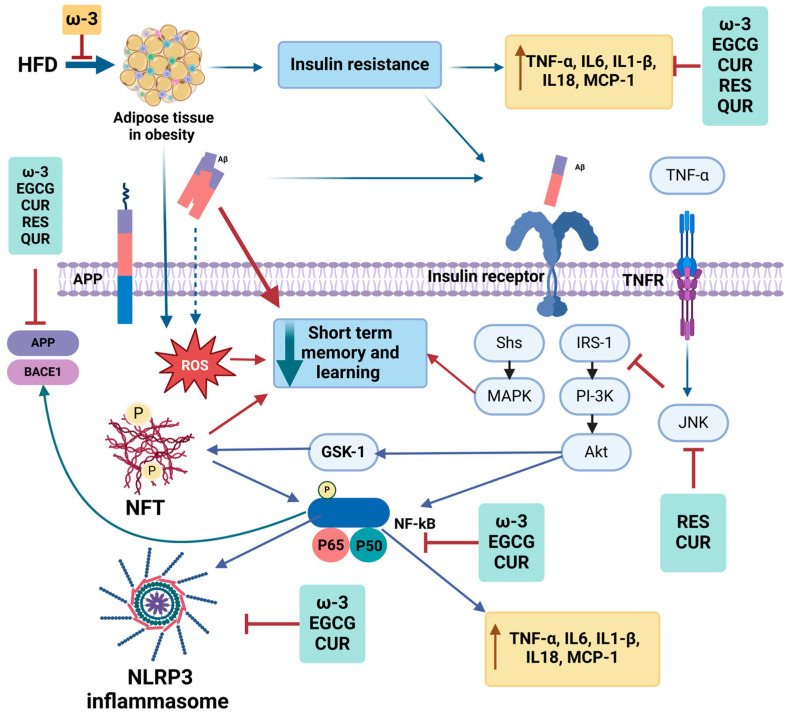
Summary of the mechanistic effects of dietary ω-3 fatty acids and polyphenols on the amyloidogenic pathway. In the context of obesity, IR, and the presence of Aβ, inflammatory pathways are upregulated. ω-3 fatty acids and polyphenols intervene in these pathways from various angles, offering potential modulation and therapeutic avenues. Akt, Ak strain transforming; APP, amyloid precursor protein; Aβ, amyloid-beta; BACE1, beta-secretase 1; CUR, curcumin; EGCG, epigallocatechin-3-gallate; GSK-3, glycogen synthase kinase 3; HFD, high-fat diet; IL18, interleukin 18; IL1β, interleukin 1 beta; IL6, interleukin 6; IRS-1, insulin receptor substrate; JNK, c-Jun N-terminal kinase; MAPK, mitogen-activated protein kinase; MCP-1, monocyte chemoattractant protein-1; NFT, neurofibrillary tangle; NF-κB, nuclear factor kappa; P, phosphorylated; QUR, quercetin; RES, resveratrol; ROS, reactive oxygen species; TNFα, tumor necrosis factor-alpha; TNFR, tumor necrosis factor receptor; ω-3, omega-3 fatty acids.

Building on the connection between metabolic dysfunction, adipokine signaling, and AD, dietary interventions emerge as a promising strategy to mitigate disease progression. Given the role of IR, inflammation, and oxidative stress in AD pathology, bioactive food compounds, such as polyphenols and omega-3 (ω-3) fatty acids, offer potential neuroprotective effects. These compounds may influence Aβ clearance, tau phosphorylation, and metabolic regulation, making them valuable for both prevention and treatment (in vivo studies are included in Table 5, and mechanistic effects are summarized in Figure 2). In this review, we highlight the effects of the polyphenols resveratrol, epigallocatechin-3-gallate (EGCG), curcumin, and quercetin and ω-3 fatty acids in AD.

### 1.6. Resveratrol

Resveratrol (trans-3,40,5-trihydroxystilbene) is a polyphenol and biologically active component found in red wine, grapes, nuts, and berries [99]. As a compound with anti-inflammatory, antioxidant, and anti-diabetic properties, resveratrol has attracted attention due to its effectiveness in treating metabolic disorders and ameliorating cognitive decline. Numerous studies have demonstrated that resveratrol inhibits neuroinflammation [100,101,102,103]. In a randomized double-blind study with 119 AD participants, dietary supplementation with 500 mg/day of resveratrol for 52 weeks prevented the loss of neurons in the brain, indicating that resveratrol could penetrate the BBB, affecting cognition and memory [104,105]. A large nationwide clinical trial investigating long-term, high-dose resveratrol supplementation in individuals with mild to moderate AD enrolled 119 participants over one year. The study reported that resveratrol supplementation (1g orally twice daily) attenuated progressive declines in CSF Aβ40 levels compared to a placebo [106]. By contrast, a randomized, placebo-controlled trial evaluated the safety and efficacy of a low-dose resveratrol (5 mg orally twice daily) treatment in 39 patients with mild to moderate AD over one year. While the resveratrol group showed less cognitive and functional decline than the control group, none of the differences reached statistical significance [107]. High-dose resveratrol activates SIRT1 despite its low bioavailability but high bioactivity [108,109], whereas low-dose intervention focuses on metabolic enhancement via antioxidant effects, mimicking dietary intake levels. In vitro studies showed that resveratrol decreased IR, intracellular Aβ peptides, and reduced tau phosphorylation by inactivating GSK3 to defend against aging and AD processes [110], through the silent information regulator 1 (SIRT1), AMP-activated protein kinase (AMPK), NLRP3 inflammasome, and NF-kB pathways [99,101]. Further, microglia treatment with resveratrol reduced the pro inflammatory markers and increased antioxidant gene expression [111]. Through these pathways, resveratrol alleviates neuronal oxidative damage, protects neurons, and enhances the ability of astrocytes to clear Aβ. This action occurs during the early stages of AD, delaying the formation of amyloid deposits [99]. Wen et al. found that the neuroprotective effects of resveratrol are reflected by the inhibition of Aβ-induced neurotoxicity mediated by efficient decreases in intracellular reactive oxygen species (ROS) via the PI3K/Akt signaling pathway in rat primary cortex neurons [112]. Recently, resveratrol has been shown to activate the PI3K/Akt insulin pathway, leading to the inhibition of GSK-3β in neurotoxic conditions. Furthermore, it has modulated the expression of TAU, RELN, metalloproteases, and their inhibitors, linked to AD pathology, thereby enhancing its neuroprotective properties [113]. Vingtdeux et al. observed the activation of AMPK and a reduction in plaques in the cortex after 15 weeks of resveratrol supplementation in APP/PS1 transgenic mice [114]. Also, it has been shown that both aerobic exercise and resveratrol supplementation reversed the decrease in AMPK/PGC-1α/SIRT1 expression in the hippocampus of AD rats, suggesting their potential to mitigate AD complications through the modulation of this critical signaling pathway [115]. Resveratrol exhibits promising neuroprotective effects by targeting multiple pathways, including inflammation, oxidative stress, and Aβ deposition, suggesting its potential in the treatment and prevention of AD.

### 1.7. Epigallocatechin-3-Gallate (EGCG)

EGCG is a major polyphenol isolated from green tea that has neuroprotective effects against Aβ neurotoxicity [116]. A double-blind, placebo-controlled, phase 2 clinical trial investigated the effects of EGCG combined with cognitive training on 84 young adults with Down syndrome, who have a high risk of AD, over a year. Although most cognitive tests showed no significant differences, participants in the EGCG (9 mg/kg per day) group exhibited improvements in visual recognition memory, inhibitory control, and adaptive behavior [117]. In the aspect of mental health, an 8-week double-blind, placebo-controlled clinical trial that investigated EGCG as an adjunct to antipsychotic medication in 34 participants with schizophrenia reported that EGCG (300 mg orally twice daily) did not provide additional benefits for psychiatric symptoms or inflammatory markers compared to a placebo, suggesting no therapeutic effect of EGCG in this pilot study [118]. Related to AD, a phase 2 clinical trial conducted at Charité University in Berlin (Clinical trial ID: 2009-009656-20) evaluated the effects of 18 months of EGCG treatment in early-stage AD patients; however, the results have not been published, leaving its efficacy in human subjects undetermined. Evidence has shown that the limited effects in the clinical studies of EGCG for mental diseases are primarily attributable to its poor bioavailability, rapid metabolism, and limited brain penetration [119]. To address these limitations, nanotechnology-based delivery systems, such as nanoencapsulation and lipid nanoparticles, have been developed to enhance EGCG’s stability, absorption, and targeted delivery to the brain, thereby potentially improving its efficacy in AD treatment [120]. Nano polymer carriers of EGCG, which could cross the BBB, enhanced synaptogenesis, memory, and learning processes by reducing Aβ and BACE1 protein expression in the hippocampus of APP/PS1 mice, a mouse model of AD [121]. The main effect of EGCG is through the inhibition of microglial activation by affecting the role of TNFα-inducing MAPK signaling pathways, and as a result, it can suppress an Aβ-induced increase [122]. This was shown in an in vitro study, where EGCG suppressed TNFα and induced nitric oxide synthase in microglia [122].

Studies have described that EGCG administrated intraperitoneally (20 mg/kg) daily for 60 days [123] decreased Aβ levels and plaque formation in the brain of APP/PS1 Tg mice via decreasing the expression of APP and Aβ in the hippocampus region, confirming the protective effect of EGCG in AD [124]. Similarly, Lin et al. showed that EGCG prevents the formation of Aβ plaques by the inhibition of APP proteolysis and by affecting GSK3 activation, which could have a preventive effect in tau phosphorylation [125]. A dose of 25 mg/kg, significantly improved spatial memory deficits and modulated hippocampal markers, including APP and brain-derived BDNF, in an AD rat model [126], suggesting EGCG’s potential therapeutic effects for AD.

### 1.8. Curcumin

Curcumin is a polyphenol that is the main bioactive component of the *Curcuma longa* rhizome known as turmeric, which is used as a spice, food color, and as a preventive or therapeutic remedy in Asian countries and cultures [127,128,129]. Studies have shown that curcumin has a variety of beneficial properties including antioxidant and anti-inflammatory actions by acting on different molecular targets, including transcription factors, growth factors, receptors, cytokines, and enzymes [130]. Curcumin also has effects on chronic age-related diseases, especially neurodegenerative diseases [131]. Both curcumin and its analogs have been applied in the clinical interventions of AD [132,133,134]. Curcumin’s poor bioavailability limits its therapeutic potential. To overcome these challenges, bioavailable formulations such as nanoparticle-based delivery systems, liposomal curcumin, phospholipid complexes (like Meriva^®^), and colloidal dispersions (like Theracurmin^®^) have been developed to enhance stability, absorption, and brain penetration, improving its efficacy in neurodegenerative diseases. Clinical studies have explored curcumin as a potential treatment for AD-related biomarkers. A randomized, placebo-controlled trial examined the effects of bioavailable curcumin on memory and brain pathology in 40 non-demented adults over 18 months. Participants receiving curcumin (90 mg twice daily, n = 21) showed significant improvements in memory and attention. The study revealed reduced amyloid and tau binding in the amygdala with curcumin, while the placebo group showed increased hypothalamic binding, suggesting curcumin’s cognitive and neuroprotective potential [135]. In another study, Di Silvestro et al. conducted a 4-week, placebo-controlled study of healthy middle-aged adults to determine the effects of lipidated curcumin (80 mg/day). Compared to the placebo, curcumin intervention significantly decreased the plasma levels of triglycerides and Aβ protein, while increasing antioxidant activity (plasma catalase, nitric oxide) [136].

In animal studies, a male Tg 5xFAD mouse model treated with 150 mg/kg/day of curcumin was protected from synaptic degradation and had improved spatial learning and memory outcomes compared to the placebo group [137]. Curcumin has been considered as a highly pleiotropic molecule regulating numerous transcription factors, including inhibiting the activation of NF-κB and inhibiting BACE1 [137,138]. Also, studies suggest that curcumin decreased Aβ production through inactivating GSK-3β and inhibiting the abnormal excessive phosphorylation of tau [139]. Several mouse studies confirmed the effect of curcumin on reduced Aβ levels in the hippocampus by increasing Aβ clearance and IDE expression [140,141,142,143,144]. Further, APP/PS1 transgenic mice supplemented with 200 and 400 mg/kg/day curcumin for 6 months had an increased expression of PI3K/Akt and downregulated IR in the hippocampus, accompanied with improved insulin signaling, memory, and learning [141]. Similar results were reported by Kanti et al. in the AD rat model [145].

### 1.9. Quercetin

Quercetin, a flavanol with several health benefits, altered several signaling pathways in AD [146]. Quercetin has widespread availability amongst dietary sources such as fruits and vegetables and is also found in barks and rinds [147]. The anti-aggregation effect of quercetin has been demonstrated by its ability to destabilize the oligomeric species of misfolded proteins and inhibit the formation of Aβ fibrils [148]. Quercetin also reduced inflammation in astrocytes and neuronal cultures by inhibiting IL-1β, IL-6, and IL-8 [149]. Quercetin was established to exert beneficial effects in diabetes using both in vitro and in vivo studies. There is a lack of published clinical trials evaluating quercetin’s efficacy in human subjects with AD. The limited bioavailability and poor blood–brain barrier penetration of quercetin pose significant challenges to its therapeutic application in neurodegenerative diseases [150]. Related to aging and mental disorders, an ongoing pilot clinical study by the Washington University School of Medicine (Clinical trial ID: NCT05838560- 202302203) aims to evaluate the safety and feasibility of dasatinib + quercetin in aging-related schizophrenia and depression. Up to 40 participants will be enrolled for one year, receiving eight doses of dasatinib (100 mg) and quercetin (1250 mg) over four weeks. The study will assess changes in the senescence-associated secretory phenotype, neuropsychological function, clinical symptoms, and brain-aging markers via MRI and blood draws at multiple time points. On the other hand, various pre-clinical studies have been conducted. In streptozocin-induced diabetic rats, quercetin reduced blood glucose levels, increased insulin levels, and improved memory and learning functions [151,152]. Some studies indicated the effect of quercetin in improving cognition and Aβ in AD mouse models. It was reported that quercetin-3-O-glucuronide, a quercetin analog, has protective effects against AD by reducing Aβ generation, reducing Aβ oligomerization, and promoting neuroplasticity processes in the Tg2576 mouse model of AD [153]. Moreno et al. used nano-capsulated quercetin in the male SAMP8 mouse model of AD and observed improved cognitive deficits and reduced memory impairments [154]. Zhang et al. used a 5xFAD mouse model of AD and fed them 500 mg/kg quercetin for 10 days and reported a reduction in insoluble Aβ levels and increases in brain apolipoprotein E [155]. In in vitro studies, quercetin inhibited BACE1 and prevented Aβ formation [156]. Also, quercetin showed GSK3 inhibitory activity and consequently inhibited the hyperphosphorylation of tau [156]. Hu et al. indicated that quercetin supplementation in diabetic mice increased the protein expression of SIRT1 and decreased the expression of inflammation-related proteins, including NLRP3, and cleaved caspase-1 and pro-inflammatory cytokines such as IL-1β and IL-18, suggesting that quercetin may be important in the treatment of AD [157].

### 1.10. Omega-3 Polyunsaturated Fatty Acids

Fatty acids serve as a substrate for energy as well as an integral component of the brain membranes [158]. Polyunsaturated fatty acids (PUFAs) play an important role in inflammation, oxidative stress, and neuronal functionality by modulating membrane phospholipids and second messengers [159]. Fish oil contains long-chain ω-3 PUFAs that include eicosapentaenoic acid (EPA, 20:5 ω-3) and docosahexaenoic acid (DHA, 22:6 ω-3) [160]. Fish oil is known for its anti-inflammatory, triglyceride-lowering, and neuroprotective properties against dementia [161,162]. Also, limited studies indicate the anti-diabetic effect of fish oil supplementation and reduction in IR markers [163,164]. Lower levels of DHA in the serum, as well as high dietary intake ratios of ω-6/ω-3 fatty acids, have been linked to both metabolic dysfunction and cognitive impairments in humans [165].

Our lab previously reported that EPA reduced systemic inflammation, obesity, and IR in diet-induced obese (DIO) mice [166,167]. Further, EPA supplementation in APPswe/PS1dE9 mice reduced the circulating Aβ 40 in male mice [168]. Thus, it is plausible that ω-3 fatty-acid-rich diets may impact AD pathogenesis and reduce the risk of AD. However, the underlying mechanisms are still unclear [169]. Based on epidemiological findings, the deficiency of ω-3 fatty acids, EPA, and DHA, and a high ratio of ω-6/ω-3 fatty acids are associated with mild depression and AD in humans [170]. In line with this, in 1999, Kyle et al. suggested that decreased DHA levels in the plasma of elderly individuals are predictive of the development of AD after 10 years [171]. Several observational studies evaluated the association between the consumption of fish or ω-3 PUFAs and cognitive performance.

Heude et al., in a prospective cohort study, measured the fatty acid content of the erythrocyte membrane in healthy individuals and conducted cognitive assessments for 4 years and revealed that higher ω-3 fatty acid levels in the blood were associated with a 41% less cognitive decline; the results were statistically significant for DHA but not for EPA levels [172]. A large community study examined the impact of ω-3 fatty acid intake on age-related cognitive decline among older adults. Morris et al. reported that, over six years of follow-up, individuals consuming fish at least once weekly were associated with a reduction in cognitive decline of 10–13% compared to those eating fish less frequently [173]. However, ω-3 PUFAs showed little association with cognitive change. A 5-year prospective study (Zutphen Elderly Study) measured fish consumption based on food frequency questionnaires and the levels of DHA and EPA from both fish and other sources and concluded that a moderate intake of EPA+DHA might postpone cognitive decline in older adults [174]. Also, several clinical trials have been conducted in patients with AD by involving ω-3 fatty acid supplementation. A double-blind, placebo-controlled study investigated whether ω-3 fatty acids alter the fatty acid profile in the CSF of patients with mild to moderate AD. Among the 33 participants, individuals who were supplemented with 2.3 g of ω-3 fatty acids, high in DHA, daily for 6 or 12 months, successfully increased the CSF levels of EPA, DHA, and total ω-3 PUFAs, while the placebo group showed no changes, suggesting the BBB crossing of these fatty acids is a potential link between DHA- and AD-related pathology [175].

In the pre-clinical aspect, studies also showed that fish oil inhibits β- and γ-secretase activity and thereby reduces amyloidogenic cleavage of APP and decreases Aβ in the female APPswe/PS1dE9 transgenic mouse model [176]. The levels of ω-3 PUFAs were negatively correlated with the levels of inflammatory cytokines IL-1β and IL-6; however, the cognitive decline was the same as the placebo group [175,177].

Some mechanisms are proposed for the effect of EPA and DHA in AD. EPA and DHA enhance microglial phagocytosis and thus can help with Aβ deposit clearance [178]. Further, fish oil also reduces pro-inflammatory cytokine TNFα and shifts the microglial phenotype from pro-inflammatory M1 to anti-inflammatory M2 [178]. DHA, which is more abundant in the brain, is better studied than EPA in experimental AD models, especially at cellular and molecular levels. In the 3xTg-AD mouse model and human neuronal-glial cells, neuroprotectin D1 stereoselective mediator derived from DHA suppressed the Aβ42 peptide by downregulating BACE1 and activating α-secretase and upregulating sAPPα, thus shifting the cleavage of βAPP from the amyloidogenic to the non-amyloidogenic pathway [179]. In cultured hippocampal neurons, DHA inhibited the Aβ oligomer-induced JNK pathway and further inhibited tau phosphorylation via the effect on GSK-3. This benefit of DHA is also reported in a mouse model of AD, and the inhibition of tau phosphorylation was associated with improved animal cognitive performance [180,181].

In summary, as shown in Figure 2, a high-fat diet leads to obesity and inflammatory adipose tissue. Studies have demonstrated that ω-3 fatty acids effectively reduce the adipose tissue inflammation associated with obesity. However, obesity can contribute to IR over time, further increasing inflammatory markers such as TNF-α, IL-6, IL-1β, IL-18, and MCP-1. Compounds such as ω-3 fatty acids, EGCG, curcumin, resveratrol, and quercetin have been shown to systematically reduce these inflammatory markers as we discussed in this review. Additionally, obesity and IR interact with the amyloid-beta pathway, promoting inflammation and oxidative stress. In the brain, IR activates TNF receptors, leading to increased TNF-α levels and the subsequent activation of the JNK, IRS-1, PI3K/AKT, and MAPK pathways. Notably, resveratrol and curcumin can inhibit JNK pathway activation, while ω-3 fatty acids, EGCG, and curcumin suppress NF-κB expression, thereby reducing neuroinflammation. Furthermore, these bioactive compounds mitigate NLRP3 inflammasome activation and downregulate APP and BACE1 expression, key components of the AD pathway.

**Table 5:** Polyphenols and omega-3 (ω-3) fatty acid supplementation in AD animal models.

AD Model/Sex/Age	Intervention	Findings	Ref
Polyphenols			
Wistar rats- STZ/-/8–10 months	Aβ hippocampal injection -Resveratrol: 25 mg/kg BW	↑ SIRT1 expression, MDA, ↓ Memory impairment, GSH, SOD, IL-1β, IL-16	[182]
B6/male/8 weeks	Resveratrol: 100 mg/kg BW and Metformin 250 mg/kg BW	↑ AMPK phosphorylation, mTOR activation by resveratrol and combo ↓ BDNF	[183]
Tg2576 male AD mice and hUC-MSCs transplanted mice/-/5 months	Resveratrol/200 mg/kg BW	Shower synergistic effects in neuroprotection ↓ SIRT1, neural apoptosis ↑ p53, p21, neurogenesis, cognition	[184]
Tg19959/ male/4 months	Resveratrol: 300 mg/kg BW	↓ Reduction in plaque counts and plaque burden in medial cortex, GSH	[105]
3xTg-AD/male/7 months	Resveratrol: 481 mg/kg BW and exercise training	↓ NF-κB, GFAP, PARP, Aβ, BACE1 by RES or combo ↑ BDNF, NGF, synaptophysin, PSD-95 RES or combo	[185]
Tg6799/male/6 months	Resveratrol: 60 mg/kg BW	↓ Cognitive impairment, Aβ42, BACE1, APP No effect on SIRT1	[186]
5xFAD/male/10 months	Trans-resveratrol 1 g/kg BW and HFD	↓ Cognitive impairment, APP, tau, BACE1, and Aβ plaques compared to HFD	[187]
Tg2576/male and female/14 months	EGCG: 50 mg/kg BW	↓ Aβ pathology, cognitive impairments	[188]
SAMP8/-/-	EGCG: 5 and 15 mg/kg BW	↓ Aβ1–42 accumulations, tau phosphorylation, and BACE-1	[189]
Sprague-Dawley (SD) rats injected with Aβ/male/-	EGCG: 100, 250 and 625 mg/kg BW	↓ Aβ, tau phosphorylation, BACE-1 ↑ Learning and memory	[190]
APP/PS1/-/6 months	EGCG: 50 mg/kg BW	↓ Aβ, Iba ↑ synapsin-1, synaptophysin, PSD93 and GluR1, IL-10 and IL-13	[191]
Swiss Albino injected with Aβ/female/18–22 months	Free curcumin: 50 mg/kg BW, Lipid core nano capsuled curcumin: 10 mg/kg	↓ Aβ, NF-κB, TNFα, IL-1β, IL-6, IFN-γ Similar results in the high dose and nanocapsule low dose	[192]
3xTg-AD/-/16 months	Quercetin 100 mg/kg BW	↓ Aβ, and tau hyperphosphorylation ↑ cognitive function	[193]
SAMP8 mice/male/7 months	Quercetin-loaded nanoparticles: 25 mg/kg BW	↑ cognition and memory impairments	[154]
3xTg/-/21–24 months	Quercetin 25 mg/kg BW	↓ Aβ, tau level, GFAP ↑ spinal learning	[194]
ω-3 fatty acids			
SAMP8/male/12 months	EPA and DHA 10 g/kg BW	DHA and EPA ↓ PS1 and BACE1, soluble Aβ40 DHA ↓ cognitive impairment	[195]
B6 /male/2.5 months	Lysophosphatidylcholine-EPA for 2 weeks	↑ the amount of EPA and DHA in brain ↓ BDNF, CREB, and 5-HT1A, TNFα ↑ phosphorylation of CREB	[196]
SAMP8/male/9 months	200 mg/kg BW of DHA, 200 mg/kg BW of EPA Oral gavage	↓ p-JNK, PHF-1 by DHA	[197]
APP/PS1/male and female/18 months	HFD supplemented with 36 g/kg BW EPA	↓ Aβ-40 compared to HFD in the serum of the male group, ↓ serum leptin and ↑ serum adiponectin	[168]

BW: body weight; MDA: malondialdehyde; GSH: glutathione; SOD: superoxide dismutase; BDNF: brain-derived neurotrophic factor; GFAP: glial fibrillary acidic protein; PARP: poly (ADP-Ribose) polymerase; CREB: cAMP response element-binding protein; PHF-1: paired helical filament-1. ↑ increased; ↓ decreased.

## 2. Conclusions

AD is a prevalent and growing neurodegenerative disease lacking a definitive treatment or prevention strategy. Metabolic dysfunctions like obesity and diabetes are risk factors for cognitive dysfunction. Obesity-induced IR is associated with inflammation and adipokine dysfunction, which can increase Aβ aggregation and tau phosphorylation in the brain and further lead to neurodegeneration and memory and learning defects. Significantly, the buildup of Aβ exacerbates both inflammatory and insulin signaling pathways. Unfortunately, in symptomatic AD, Aβ clearance fails to outweigh the formation of Aβ plaques. As a result, apoptosis and neuronal death prevail in various brain regions, leading to a cognitive dysfunction in affected patients.

As important components of healthy diets, the selected bioactive compounds that may exert beneficial effects in AD have been reviewed in this paper.

Pre-clinical studies indicate the protective effects of polyphenol and ω-3 fatty acids interventions in obesity, IR, and AD. However, existing clinical trials investigating the bioactive compounds we discussed here, including resveratrol, EGCG, curcumin, quercetin, and ω-3 fatty acids, in AD and cognitive decline are limited with inconsistent outcomes. Indeed, many population and clinical studies have small enrollments, which reduces statistical power. Moreover, subject heterogeneity and variation across populations, including differences in the age, baseline cognitive status, methods used for these measurements, comorbidities, and genetic factors (e.g., APOEε4 status) of patients may influence their response to treatments. Additionally, short follow-up durations often fail to capture the long-term cognitive or neuroprotective effects of the compounds tested. There are also limitations related to the design of dietary interventions with further variability and discrepancies in dosage, bioavailability, and formulations. Some studies used native compounds, while others used high doses of pure chemicals with varying bioavailable forms, further contributing to the inconsistency in outcomes. Addressing these limitations with larger, longer, and more rigorously designed double-blind, randomized clinical trials that also assess sex differences will be essential to determining the true efficacy of these bioactive compounds in AD prevention and treatment.

## Abbreviations

The following abbreviations are used in this manuscript:

AD	Alzheimer’s disease
AKT	Protein kinase B
APP	Amyloid precursor protein
Aβ	Amyloid-beta
BACE1	Beta-secretase 1
BBB	Blood–brain barrier
CUR	Curcumin
DIO	Diet-induced obese
EGCG	Epigallocatechin-3-gallate
FDA	Food and Drug Administration
GSK3	Glycogen synthase kinase 3
HFD	High-fat diet
IDE	Insulin-degrading enzyme
IL18	Interleukin 18
IL1β	Interleukin 1 beta
IL6	Interleukin 6
IR	Insulin resistance
IRS-1	Insulin receptor substrate
JNK	c-Jun N-terminal kinase
LRP	Low-density lipoprotein receptor-related protein 1
MAPK	Mitogen-activated protein kinase
MCP-1	Monocyte chemoattractant protein-1
NFT	Neurofibrillary tangle
NF-kB	Nuclear factor kappa
NLRP3	NOD-, LRR-, and pyrin domain-containing protein 3
PI3K	Phosphatidylinositol 3-kinase
PUFAs	Polyunsaturated fatty acids
QUR	Quercetin
RES	Resveratrol
ROS	Reactive oxygen species
SIRT1	Silent information regulator 1
T2DM	Type two diabetes mellitus
TNFα	Tumor necrosis factor-alpha
TNFR	Tumor necrosis factor receptor
ω-3	Omega-3 fatty acid
